# Malformation anorectale et complexe sphinctérien anorectal

**DOI:** 10.11604/pamj.2016.25.195.10870

**Published:** 2016-11-25

**Authors:** Amira Ayachi, Mechaal Mourali

**Affiliations:** 1 Service de Gynécologie et Obstétrique, CHU Bougatfa, Bizerte, Faculté de Médecine de Tunis, Université El Manar, Tunisie

**Keywords:** Diagnostic anténatal, malformation anorectale, défaut de fermeture du tube neural, Prenatal diagnosis, anorectal malformations, neural tube defect

## Image en médecine

Il s’agit d’une patiente adressée à notre unité de diagnostic anténatal pour suspicion de spina bifida à un terme de 22 semaines d’aménorrhée. Le bilan morphologique trouvait un rachischisis associé à un Chiari II, un rein unique et une malformation anorectale. L’absence de visualisation du complexe sphinctérien anorectal (CSAR) à l’échographie faisait suspecter une malformation anorectale haute (A, B, C). L’examen échographique ne montrait ni anomalies des membres, ni cardiopathies, ni signes en faveurs d’une atrésie de l’œsophage. Un caryotype sur liquide amniotique a été réalisé et le résultat ne montrait pas d’anomalies (46, XY). Une interruption de la grossesse a été demandée par les parents après explication du pronostic fœtal montrant une absence d’anus (D). L’examen fœtopathologique permet de confirmer la nature haute ou basse de la malformation anorectale par l’absence de CSAR et les anomalies associées.

**Figure 1:**
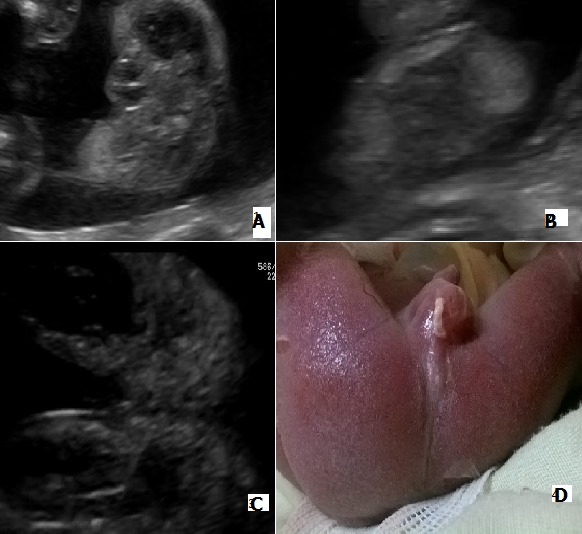
A) absence de visualisation du complexe sphinctérien anorectal; B) image échogène linéaire au niveau du périnée; C) ébauche de pli fessier; D) absence d’anus

